# Can zoledronic acid reduce the risk of cage subsidence after oblique lumbar interbody fusion combined with bilateral pedicle screw fixation in the elderly population? A retrospective study

**DOI:** 10.1186/s13018-024-04828-3

**Published:** 2024-06-08

**Authors:** Cong Jin, JinXiang Shang, Xi Chen, Jiewen Zheng, Wei He, Lei He, Weiqi Han

**Affiliations:** 1 Department of Orthopedics, Shaoxing People’s Hospital, Zhongxing North Road, Shaoxing, Zhejiang 312000 China; 2 Department of Orthopedics, Affiliated Hospital of Shaoxing University, Shaoxing, Zhejiang 312000 China; 3 School of Medicine, Shaoxing University, Shaoxing, Zhejiang 312000 China

**Keywords:** Zoledronic acid, Risk, Spinal Fusion

## Abstract

**Background:**

The objective of this study was to evaluate the potential of zoledronic acid for reducing the incidence of cage subsidence and enhancing interbody fusion rates following oblique lumbar interbody fusion (OLIF) surgery, particularly as the first reported evidence of the role of zoledronic acid combined with OLIF.

**Methods:**

A retrospective analysis was conducted on data from 108 elderly patients treated for degenerative lumbar diseases using OLIF combined with bilateral pedicle screw fixation from January 2018 to December 2021. Patients were divided into the zoledronic acid (ZOL) group (43 patients, 67 surgical segments) and the control group (65 patients, 86 surgical segments). A comparative analysis of the radiographic and clinical outcomes between the groups was performed, employing univariate and multivariate regression analyses to explore the relationships between cage subsidence and the independent variables.

**Results:**

Radiographic outcomes, including anterior height, posterior height, disc height, coronal disc angle, foraminal height, and lumbar lordosis, were not significantly different between the two groups. Similarly, no statistically significant differences were noted in the back visual analog scale (VAS) scores and Oswestry Disability Index (ODI) scores between the groups. However, at the 1-year follow-up, the leg VAS score was lower in the ZOL group than in the control group (*P* = 0.028). The ZOL group demonstrated a notably lower cage subsidence rate (20.9%) than did the control group (43.0%) (*P* < 0.001). There was no significant difference in the interbody fusion rate between the ZOL group (93.0%) and the control group (90.8%). Non-use of zoledronic acid emerged as an independent risk factor for cage subsidence (OR = 6.047, *P* = 0.003), along with lower bone mineral density, lower postoperative anterior height, and concave endplate morphology. The model exhibited robust discriminative performance, with an area under the curve (AUC) of 0.872.

**Conclusion:**

The administration of zoledronic acid mitigates the risk of cage subsidence following OLIF combined with bilateral pedicle screw fixation in elderly patients; however, it does not improve the interbody fusion rate.

**Supplementary Information:**

The online version contains supplementary material available at 10.1186/s13018-024-04828-3.

## Background

Oblique lumbar interbody fusion (OLIF) is a minimally invasive lumbar fusion technique that involves retroperitoneal access between the psoas muscle and artery, with oblique cage insertion to restore intervertebral height and enlarge the spinal canal and intervertebral foramina, indirectly achieving nerve decompression [1, 2]. Compared to posterior fusion techniques, OLIF minimizes damage to posterior paravertebral muscles and soft tissues, reducing surgical trauma and postoperative low back pain [3, 4]. Additionally, it diminishes nerve interference, reducing the risk of nerve injury [4], and offers a larger fusion area, facilitating interbody fusion and stability [1].

Cage subsidence, a common postoperative complication following OLIF surgery, has been extensively investigated [5]. Despite efforts to mitigate its risk through OLIF combined with posterior pedicle screw fixation, its incidence remains considerable. For instance, Wen J reported a subsidence rate of 43.2% among 74 patients treated with OLIF combined with pedicle screw fixation, with rates of 38.8% for bilateral fixation and 47.4% for unilateral fixation [6]. Cage subsidence may lead to instability of the intervertebral space, delayed fusion, and even pseudoarthrosis [5]. Additionally, decreased intervertebral height due to subsidence compromises the indirect decompression effect of OLIF, potentially leading to recurrent neurological symptoms and the need for reoperation [5]. Therefore, preventive strategies against cage subsidence following OLIF surgery are imperative.

Zoledronic acid, widely used for osteoporosis treatment, inhibits osteoclasts, increases bone density, and reduces fracture risk [7]. Reports suggest that zoledronic acid reduces the risk of vertebral compression fractures and refracture in osteoporotic patients following vertebral augmentation procedures [8, 9]. Recent studies on its use in posterior lumbar fusion techniques indicate its potential to reduce cage subsidence risk [7, 10–12]. Posterior lumbar fusion can cause bone loss around the fusion site due to reduced mechanical loading and immobilization. Zoledronic acid may prevent bone loss by maintaining bone turnover and preserving bone mass, reducing the risk of cage subsidence associated with weakened bone structure [7, 12]. Additionally, by inhibiting osteoclast activity, zoledronic acid helps maintain or increase bone density [7], providing better support for the cage and reducing the risk of subsidence. However, there is currently no literature reporting on the role of zoledronic acid in combination with OLIF for treating degenerative lumbar diseases in the elderly population.

This retrospective analysis of 108 elderly patients undergoing OLIF with bilateral pedicle screw fixation aimed to assess the potential of zoledronic acid in reducing cage subsidence and enhancing fusion rates. Patients were divided based on postoperative zoledronic acid use, and radiographic and clinical outcomes were compared, with multivariate analysis to investigate relationships between cage subsidence and independent variables. This study provides initial evidence of the role of zoledronic acid in OLIF surgery for degenerative lumbar diseases in the elderly population.

## Methods

### Study design

This was a retrospective study conducted at Shaoxing People’s Hospital that analyzed data from elderly patients treated for degenerative lumbar diseases using OLIF combined with bilateral pedicle screw fixation from January 2018 to December 2021. Patients were divided into the zoledronic acid (ZOL) group and the control group based on whether zoledronic acid was used postoperatively. In the ZOL group, patients received intravenous infusion of 5 mg of zoledronic acid within 3 days postoperatively and then again at 1 year after surgery. The radiographic and clinical outcomes of both groups were compared, and univariate and multivariate regression analyses were employed to analyze the relationships between cage subsidence and the independent variables. This study obtained approval from the Ethics Committee of Shaoxing People’s Hospital (NO2024-052-Y-01), and informed consent was obtained from all patients.

### Participants

The inclusion criterion for this study was patients aged over 50 years who underwent treatment for degenerative lumbar diseases via OLIF combined with bilateral pedicle screw fixation. The diagnoses included lumbar disc herniation, spondylolisthesis, spinal stenosis, and degenerative scoliosis. Patients were followed up for more than 1 year postoperatively. The exclusion criteria included recent (within the past 3 months) or long-term use of glucocorticoids, teriparatide, other bisphosphonate drugs, or other medications affecting bone metabolism. Patients with severe hepatic or renal dysfunction, bone tumors, or metastatic bone tumors were also excluded. Patients with serum calcium concentrations greater than 2.75 mmol/L or less than 2.0 mmol/L, as well as those with a history of spinal surgery within the past 6 months, were excluded from the study.

### Surgery and postoperative care

All surgeries were performed by the same surgical team. After successful general anesthesia induction, patients were positioned in the right lateral decubitus position with left hip flexion to alleviate tension in the psoas muscle. An oblique incision was made 5 cm ventral to the center point of the intervertebral disc, followed by dissection through the skin and subcutaneous tissue. The oblique external abdominal oblique, internal abdominal oblique, and transversus abdominis muscles were sequentially dissected bluntly along the direction of the muscle fibers. Upon reaching the retroperitoneum, the peritoneum along with the retroperitoneal fat was pushed ventrally to expose the anterior edge of the psoas muscle. The psoas muscle was retracted posteriorly at the level of the intervertebral disc, and a guide needle was placed under fluoroscopic guidance. After confirming the correct surgical segment, an OLIF working tube was installed, and the intervertebral disc was excised within the working tube. The cartilaginous endplates were managed, and an appropriately sized OLIF fusion cage (filled with allogeneic bone) was inserted. Following completion of the OLIF procedure, patients were positioned prone, and bilateral pedicle screws were placed through the Wiltse approach for fixation.

Within 48 h postoperatively, all patients had their wound drainage tubes removed, a soft brace was worn for ambulation, and routine exercises were performed to strengthen the muscles of the back and lower limbs. Throughout the follow-up period, all patients received standard treatment consisting of daily oral administration of 600 mg calcium carbonate and 0.25 µg calcitriol. Patients in the zoledronic acid group received intravenous infusion of 5 mg of zoledronic acid within 3 days postoperatively and then again at 1 year after surgery.

### Baseline data

The baseline data of all patients, including age, gender, body mass index (BMI), diagnosis, surgical segment, number of surgical segments, comorbidities such as diabetes and hypertension, and smoking and alcohol history, were obtained directly from the hospital’s electronic medical records system.

### Radiographic evaluation

**Fig. 1:**
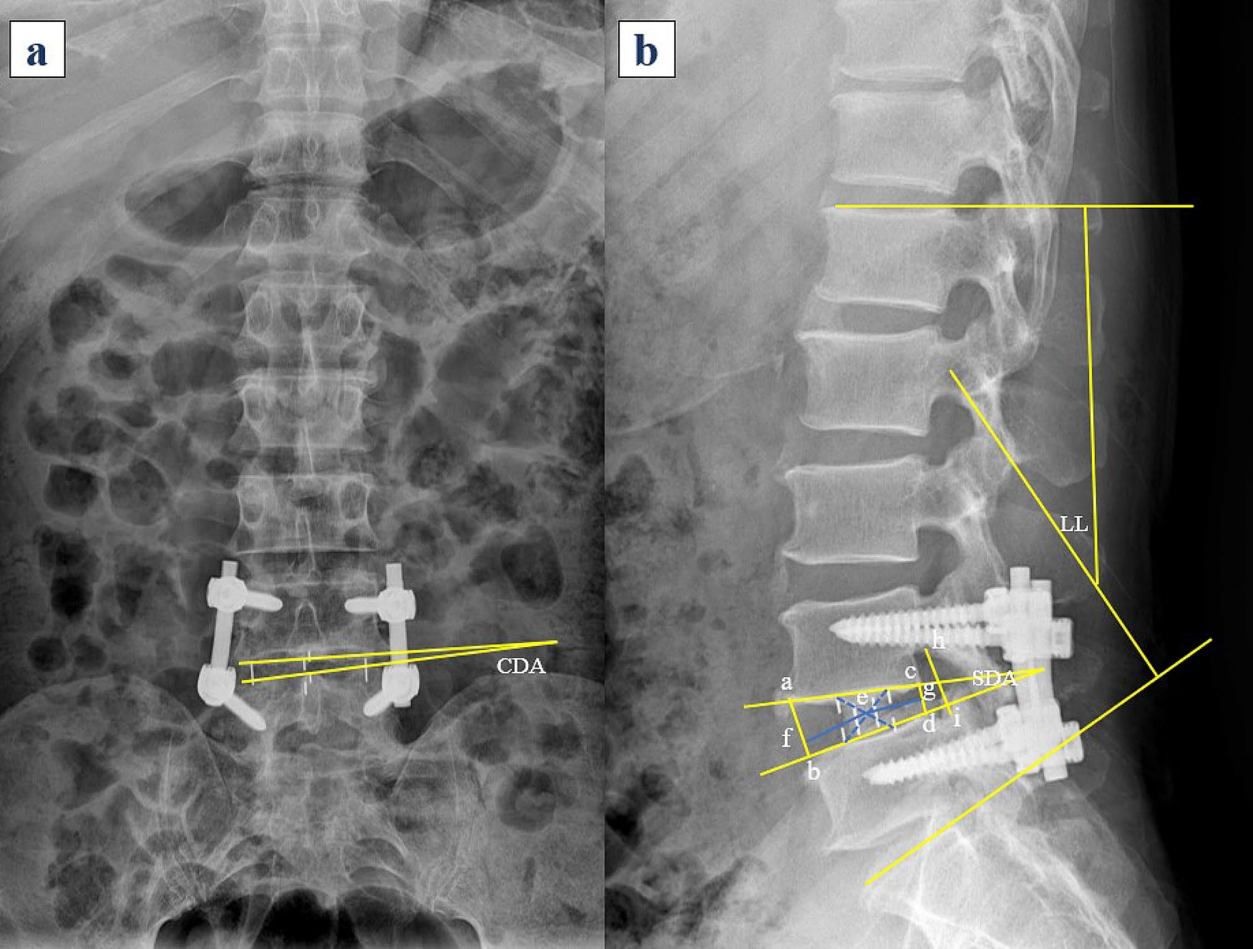
Measurement of radiological data. (**a**) The coronal disc angle (CDA) was defined as the angle between the lower endplate of the superior vertebra and the upper endplate of the inferior vertebra on anteroposterior X-ray images. (**b**) The sagittal disc angle (SDA) was defined as the angle between the lower endplate of the superior vertebra and the upper endplate of the inferior vertebra on lateral X-ray images. Line segment ab represents the anterior height (AH) of the intervertebral space, while line segment cd represents the posterior height (PH) of the intervertebral space. The disc height (DH) is defined as the average of the AH and PH. The foraminal height (FH) is defined as the distance between the highest point (**h**) of the superior pedicle and the lowest point (**i**) of the inferior pedicle. LL is defined as the angle formed by the perpendicular lines from the superior endplate of the first sacral vertebra (S1) and the superior endplate of the first lumbar vertebra (L1). Point e represents the center of the cage, and the location of the cage is defined as the ratio of the distance from the center point e to the anterior edge of the disc space (ef) to the distance from the center point e to the posterior edge of the disc space (eg). CDA: coronal disc angle; AH: anterior height; PH: posterior height; DH: disc height; SDA: sagittal disc angle; FH: foraminal height; LL: lumbar lordosis; SD: standard deviation; IQR: interquartile range

According to previous literature [13–15], radiographic evaluation encompasses measurements of the anterior height of the intervertebral space (AH), posterior height of the intervertebral space (PH), intervertebral disc height (DH), coronal intervertebral disc angle (CDA), sagittal intervertebral disc angle (SDA), foraminal height (FH), and lumbar lordosis (LL). The evaluation time points included the preoperative, postoperative, and 1-year follow-up periods. Measurements were directly conducted on X-ray anteroposterior and lateral views utilizing the electronic measurement tools provided by the Picture Archiving and Communication System (PACS V3.0, Zhejiang Rad Information Technology Company, China). The measurements were independently performed by two radiologists, and the average value was derived. The measurement methodology is described in Fig. 1.

At the 1-year follow-up, cage subsidence and interbody fusion status were evaluated for all patients. According to previous literature [3, 16, 17], cage subsidence is considered present if a cage is observed to sink into an adjacent vertebral body by more than 2 mm. The interbody fusion status was evaluated by one experienced orthopedic surgeon and one radiologist. Fusion was deemed achieved when both assessors concurrently confirmed fusion. In accordance with prior literature [7, 11], interbody fusion was required to satisfy the following criteria: (1) reconstructed CT sagittal images exhibited evident trabecular bone growth, forming bone bridges extending from the cage to the upper and lower endplates, with no radiolucent zones between the cage and endplates. (2) Continuous trabecular bone was also observed surrounding the cage, bridging the upper and lower endplates, while the intervertebral space was consistently filled with bone graft, forming trabecular bone. (3) Flexion and extension X-rays demonstrated a range of motion at the fused segment of less than 5°.

Preoperative MRI was utilized to evaluate the degree of paraspinal muscle degeneration, endplate morphology, and endplate lesions. Consistent with prior literature [18], the degree of paraspinal muscle degeneration was assessed using T2-weighted MR images at the midline transverse level of the L3 vertebra. A grade of 0 indicated normal, 1 indicated less than 10% replacement of muscle area by fat and fibrous tissue, 2 indicated replacement of muscle area between 10% and 50%, and 3 indicated replacement of muscle area by more than 50%. Endplate morphology, which can exhibit either flat or concave shapes, was evaluated on sagittal T2-weighted images. Additionally, endplate lesions, including Schmorl’s nodes and Modic changes observed on sagittal T2-weighted MR images, were documented.

Bone mineral density (BMD) values were measured using dual-energy X-ray absorptiometry (DEXA), and the average BMD was obtained from L1 to L5. The evaluation time points included preoperatively, at 6 months postoperatively, and at 1 year postoperatively.

### Operative and cage-related outcomes

Operation-related outcomes included operative time and intraoperative blood loss, while cage height and length were directly obtained from the electronic medical records system. The location of the cage was assessed on postoperative lateral X-ray images, where the cage location was defined as the ratio of the distance from the cage center to the anterior margin of the intervertebral space and the distance from the cage center to the posterior margin of the intervertebral space. The detailed measurement methods are illustrated in Fig. 1b.

### Clinical outcomes

The clinical assessments included the visual analog scale (VAS) [19] score, leg VAS score, and Oswestry Disability Index (ODI) [20]score. The evaluation time points were preoperatively, postoperatively, 3 months postoperatively, and 1 year postoperatively. The VAS score was recorded as a numerical value ranging from 0 to 10, with 0 indicating no pain and 10 indicating the worst possible pain. The ODI was used to assess the level of disability and functional impairment related to low back pain.

### Statistical analysis

Statistical analysis was performed using SPSS (version 19.0; SPSS Inc., Chicago, IL, USA) on the Windows platform. The comparison of gender, history of diabetes and hypertension, smoking status, alcohol consumption status, and cage subsidence rate between the two groups were conducted through the chi-square test. Yates’ correction was applied to assess surgical segments, diagnosis, number of surgical segments, the length of the cage, and the fusion rate between the two groups. Age was evaluated through independent sample t tests, with a normal distribution confirmed by the Shapiro‒Wilk normality test and variance homogeneity assessed by Levene’s test. Welch t tests were utilized to compare BMI between the two groups. Operative time, intraoperative blood loss, cage height, and cage location were compared between the groups using Wilcoxon tests. Two-way repeated-measures ANOVA was used to compare the VAS score, ODI score, BMD, AH, PH, DH, CDA, SDA, FH, and LL between the two groups. Furthermore, logistic regression was utilized to model the relationships between cage subsidence and the independent variables. The significance level was set at 0.05.

## Results

### Baseline data

A total of 108 patients, comprising 153 surgical segments, were included in this study, with 43 patients in the ZOL group covering 67 surgical segments and 65 patients in the control group involving 86 surgical segments. The baseline data of both groups, including age, gender, BMI, BMD, diagnosis, surgical segment, number of surgical segments, comorbidities (including diabetes and hypertension), and smoking and drinking history, were not significantly different (Table 1).

**Table 1:** Baseline data of the two groups

Characteristics	ZOL group	Control group	*P* value
N	43	65	
Age, mean ± SD	67.70 ± 9.02	67.51 ± 7.92	0.908
Gender, n (%)			0.286
Male	21 (19.4%)	25 (23.1%)	
Female	22 (20.4%)	40 (37%)	
BMI, mean ± SD	23.64 ± 2.49	24.36 ± 3.56	0.219
BMD, median (IQR)	-0.70 (-1.90, 1.05)	-1.00 (-2.60, 1.10)	0.308
Diagnosis, n (%)			0.798
Spondylolisthesis	12 (11.1%)	20 (18.5%)	
Degenerative Scoliosis	5 (4.6%)	5 (4.6%)	
Spinal stenosis	22 (20.4%)	31 (28.7%)	
Disc herniation	4 (3.7%)	9 (8.3%)	
Surgical segment, n (%)			0.106
L23	7 (4.6%)	4 (2.6%)	
L34	23 (15.0%)	21 (13.7%)	
L45	37 (24.2%)	61 (39.9%)	
Number of surgical segments, n (%)			0.305
1	23 (21.3%)	46 (42.6%)	
2	16(14.8%)	17 (15.7%)	
3	4 (3.7%)	2 (1.9%)	
Diabetes, n (%)			0.538
No	37 (34.3%)	53 (49.1%)	
Yes	6 (5.6%)	12 (11.1%)	
Hypertension, n (%)			0.907
Yes	21 (19.4%)	31 (28.7%)	
No	22 (20.4%)	34 (31.5%)	
Smoking, n (%)			0.838
No	37 (34.3%)	55 (50.9%)	
Yes	6 (5.6%)	10 (9.3%)	
Alcohol, n (%)			0.270
No	36 (33.3%)	59 (54.6%)	
Yes	7 (6.5%)	6 (5.6%)	

BMI: body mass index; BMD: bone mineral density; SD: standard deviation; IQR: interquartile range

### Radiographic outcomes

During the preoperative, postoperative, and 1-year follow-up, there were no significant differences in AH, PH, DH, CDA, FH, and LL between the two groups. Similarly, no significant differences were found in the SDA between the two groups during either the preoperative or 1-year follow-up assessments. However, the postoperative SDA in the ZOL group was significantly lower at 7.21 ± 3.55° than at 8.41 ± 3.65° in the control group (*P* < 0.05) (Table 2).

**Table 2:** Radiographic outcomes of the two groups

Characteristics	ZOL group	Control group	*P* value
AH			
Preop, mean ± SD	11.63 ± 2.97	12.44 ± 2.92	0.091
Postop, mean ± SD	15.84 ± 2.63	16.73 ± 2.99	0.057
1-year, mean ± SD	14.79 ± 2.98	15.51 ± 3.36	0.172
PH			
Preop, mean ± SD	6.99 ± 2.01	7.21 ± 1.98	0.501
Postop, mean ± SD	9.86 ± 1.99	10.25 ± 2.18	0.248
1-year, mean ± SD	9.34 ± 1.93	9.56 ± 2.03	0.498
DH			
Preop, mean ± SD	9.31 ± 2.00	9.83 ± 2.14	0.129
Postop, mean ± SD	12.85 ± 1.92	13.49 ± 2.24	0.064
1-year, mean ± SD	12.07 ± 2.06	12.53 ± 2.40	0.205
CDA			
Preop, mean ± SD	2.60 ± 2.84	2.10 ± 3.28	0.326
Postop, mean ± SD	1.31 ± 1.52	1.12 ± 1.11	0.379
1-year, mean ± SD	1.48 ± 1.57	1.22 ± 1.18	0.250
SDA			
Preop, mean ± SD	6.14 ± 4.37	7.22 ± 3.91	0.111
Postop, mean ± SD	7.21 ± 3.55	8.41 ± 3.65	**0.044**
1-year, mean ± SD	7.16 ± 3.15	8.13 ± 3.23	0.065
FH			
Preop, mean ± SD	18.18 ± 3.11	17.79 ± 2.57	0.395
Postop, mean ± SD	21.81 ± 3.51	21.45 ± 2.78	0.478
1-year, mean ± SD	21.16 ± 3.35	20.46 ± 2.97	0.173
LL			
Preop, mean ± SD	40.53 ± 14.85	44.92 ± 14.47	0.130
Postop, mean ± SD	39.12 ± 11.18	41.95 ± 12.60	0.234
1-year, mean ± SD	40.46 ± 10.31	42.65 ± 10.81	0.295

AH: anterior height; PH: posterior height; DH: disc height; CDA: coronal disc angle; SDA: sagittal disc angle; FH: foraminal height; LL: lumbar lordosis; SD: standard deviation; IQR: interquartile range

### Operative and cage-related outcomes

The operative outcomes, including operative time and intraoperative blood loss, were not significantly different between the two groups. Similarly, for cage-related outcomes, such as the height of the cage, length of the cage, and location of the cage, no statistically significant differences were detected between the two groups (Table 3).

**Table 3:** Operative and cage-related data of the two groups

Characteristics	ZOL group	Control group	*P* value
Operative time (min), median (IQR)	200 (170, 247)	197 (168, 252)	0.942
Intraoperative blood loss (ml), median (IQR)	50 (50, 100)	50 (50, 100)	0.790
Height of cage (mm), median (IQR)	12 (12, 12)	12 (12, 13)	0.588
Length of cage, n (%)			0.818
45 mm	25 (23.1%)	36 (33.3%)	
50 mm	17 (15.7%)	26 (24.1%)	
55 mm	1 (0.9%)	3 (2.8%)	
Location of cage, median (IQR)	1.12 (1.01, 1.25)	1.07 (0.85, 1.21)	0.204

SD: standard deviation; IQR: interquartile range

### Clinical outcomes and BMD

**Fig. 2:**
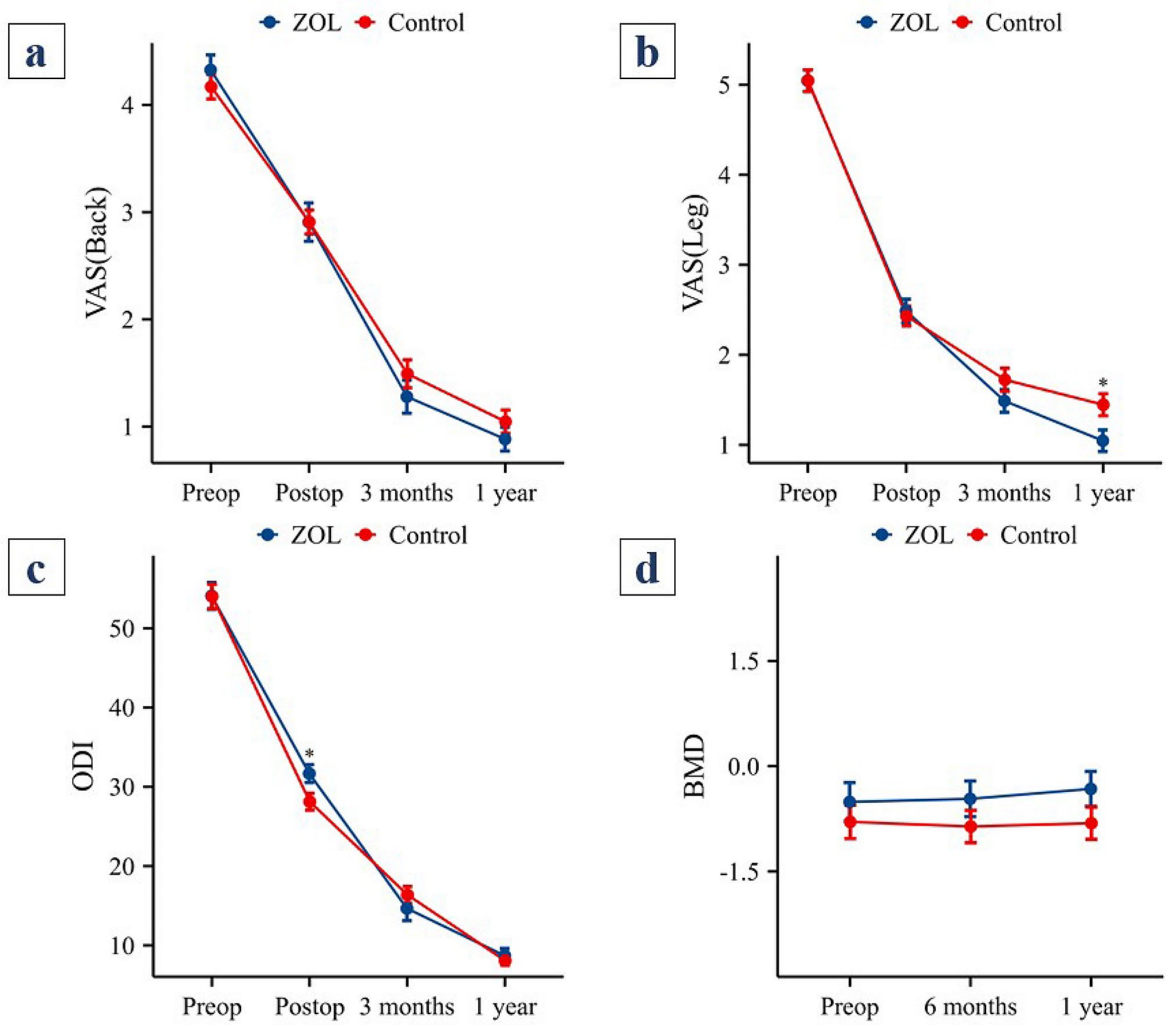
VAS scores, ODI scores and BMD of the two groups. *: *P* < 0.05 for the control group compared with the ZOL group. VAS: visual analog scale; ODI: Oswestry Disability Index; BMD: bone mineral density

During the preoperative, postoperative, 3-month postoperative, and 1-year postoperative follow-ups, no statistically significant differences were observed in the back VAS scores between the two groups. Similarly, for the leg VAS scores, no statistically significant differences were observed in the comparisons conducted before surgery, after surgery, or at the 3-month follow-up. However, at the 1-year follow-up, the leg VAS score in the control group was 1.45 ± 0.98, which was significantly greater than that in the ZOL group (1.04 ± 0.79; *P* = 0.028). Comparisons of the ODI between the two groups revealed no statistically significant differences before surgery, at the 3-month follow-up, or at the 1-year follow-up. However, postoperatively, the ODI in the ZOL group was 31.67 ± 7.38, which was significantly greater than that in the control group (28.12 ± 8.50) (*P* = 0.027) (Fig. 2a and c).

The BMD values in the ZOL group at the preoperative, 6-month postoperative, and 1-year postoperative time points were not significantly different from those in the control group. Additionally, there was no significant increase in BMD values from baseline levels in both the ZOL group and the control group at 6 months and 1 year postoperatively (*P* > 0.05) (Fig. 2d).

### Cage subsidence rates and fusion rates

**Fig. 3:**
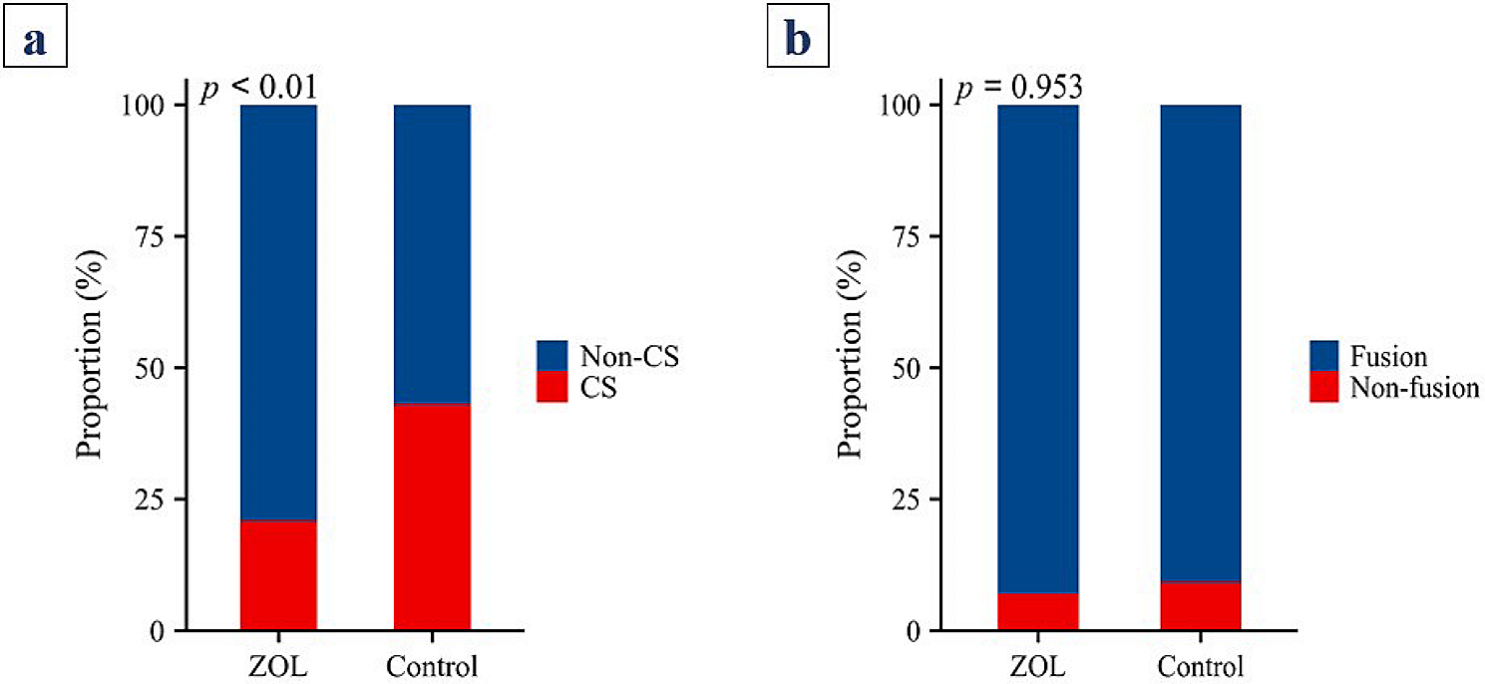
Cage subsidence rates and fusion rates of the two groups. CS: cage subsidence; non-CS: noncage subsidence

The ZOL group had 67 surgical segments, 14 of which experienced cage subsidence, resulting in a cage subsidence rate of 20.9% (14/67). In contrast, the control group had 86 surgical segments, 37 of which experienced cage subsidence, leading to a cage subsidence rate of 43.0% (37/86). A significant difference in the cage subsidence rate was detected between the two groups (*P* < 0.001) (Fig. 3a). Among the 43 patients in the ZOL group, 40 achieved interbody fusion, resulting in a fusion rate of 93.0% (40/43), whereas among the 65 patients in the control group, 59 achieved interbody fusion, resulting in a fusion rate of 90.8% (59/65). There were no significant differences in the fusion rates between the two groups (Fig. 3b).

### Risk factors for cage subsidence after OLIF combined with bilateral pedicle screw fixation

According to the univariate analysis, the risk factors associated with cage subsidence included no use of zoledronic acid (OR = 2.859, 95% CI 1.381–5.916; *P* = 0.005), lower BMD (OR = 0.522, 95% CI 0.393–0.694; *P* < 0.001), lower postoperative AH (OR = 0.863, 95% CI 0.762–0.977; *P* = 0.020), decreased postoperative FH (OR = 0.879, 95% CI 0.784–0.986; *P* = 0.028), concave endplate morphology (OR = 2.163, 95% CI 1.074–4.356; *P* = 0.031), and Modic changes (OR = 0.299, 95% CI 0.097–0.923; *P* = 0.036) (Table 4).

**Table 4:** Univariate analysis of risk factors for cage subsidence after OLIF combined with posterior fixation

Characteristics	Univariate analysis		Characteristics	Univariate analysis	
	Odds Ratio (95% CI)	*P* value		Odds Ratio (95% CI)	*P* value
Zoledronic acid			Operative time	1.002 (0.996–1.008)	0.525
No	2.859 (1.381–5.916)	**0.005**	Intraoperative blood loss	1.001 (0.997–1.005)	0.585
Yes	Reference		Preoperative AH	0.904 (0.805–1.015)	0.087
Age	1.014 (0.967–1.063)	0.570	Postoperative AH	0.863 (0.762–0.977)	**0.020**
Gender			Preoperative PH	0.958 (0.808–1.136)	0.620
Male	Reference		Postoperative PH	0.888 (0.754–1.044)	0.151
Female	1.958 (0.867–4.423)	0.106	Preoperative CDA	1.028 (0.925–1.143)	0.612
BMI	1.009 (0.892–1.141)	0.886	Postoperative CDA	1.149 (0.892–1.479)	0.282
BMD	0.522 (0.393–0.694)	**< 0.001**	Preoperative SDA	0.979 (0.902–1.062)	0.610
Diagnosis			Postoperative SDA	0.973 (0.887–1.068)	0.569
Spondylolisthesis	Reference		Preoperative FH	1.041 (0.922–1.176)	0.516
Degenerative Scoliosis	2.192 (0.515–9.332)	0.288	Postoperative FH	0.879 (0.784–0.986)	**0.028**
Spinal stenosis	0.886 (0.361–2.174)	0.791	Preoperative LL	0.984 (0.958–1.011)	0.233
Disc herniation	0.122 (0.014–1.055)	0.056	Postoperative LL	0.997 (0.965–1.030)	0.856
Number of fusion levels			Location of cage	0.925 (0.667–1.282)	0.639
1	Reference		Endplate morphology		
2	1.474 (0.628–3.457)	0.373	Flat	Reference	
3	2.000 (0.374–10.696)	0.418	Concave	2.163 (1.074–4.356)	**0.031**
Diabetes			Endplate lesion		
No	Reference		No	Reference	
Yes	1.450 (0.520–4.041)	0.477	Schmorl’s node	1.377 (0.351–5.404)	0.647
Hypertension			Modic change	0.299 (0.097–0.923)	**0.036**
Yes	Reference		Paravertebral muscle degeneration		
No	1.687 (0.765–3.725)	0.195	Grade 0	Reference	
Smoking			Grade 1	0.632 (0.139–2.867)	0.552
No	Reference		Grade 2	3.451 (0.793–15.011)	0.099
Yes	0.740 (0.237–2.310)	0.604	Grade 3	2.667 (0.434–16.390)	0.290
Alcohol			Preoperative leg VAS	0.783 (0.500–1.226)	0.284
No	Reference		Postoperative leg VAS	0.992 (0.632–1.558)	0.973
Yes	1.071 (0.325–3.531)	0.910	Preoperative back VAS	1.035 (0.677–1.583)	0.872
Height of cage	0.982 (0.714–1.353)	0.913	Postoperative back VAS	1.112 (0.754–1.639)	0.593
Length of cage			Preoperative ODI score	0.990 (0.958–1.024)	0.564
45 mm	Reference		Postoperative ODI score	1.004 (0.957–1.053)	0.874
50 mm	0.889 (0.446–1.769)	0.737			
55 mm	0.466 (0.050–4.361)	0.503			

BMI: body mass index; BMD: bone mineral density; AH: anterior height; PH: posterior height; DH: disc height; CDA: coronal disc angle; SDA: sagittal disc angle; FH: foraminal height; LL: lumbar lordosis; VAS: visual analog scale; ODI: Oswestry Disability Index

**Fig. 4:**
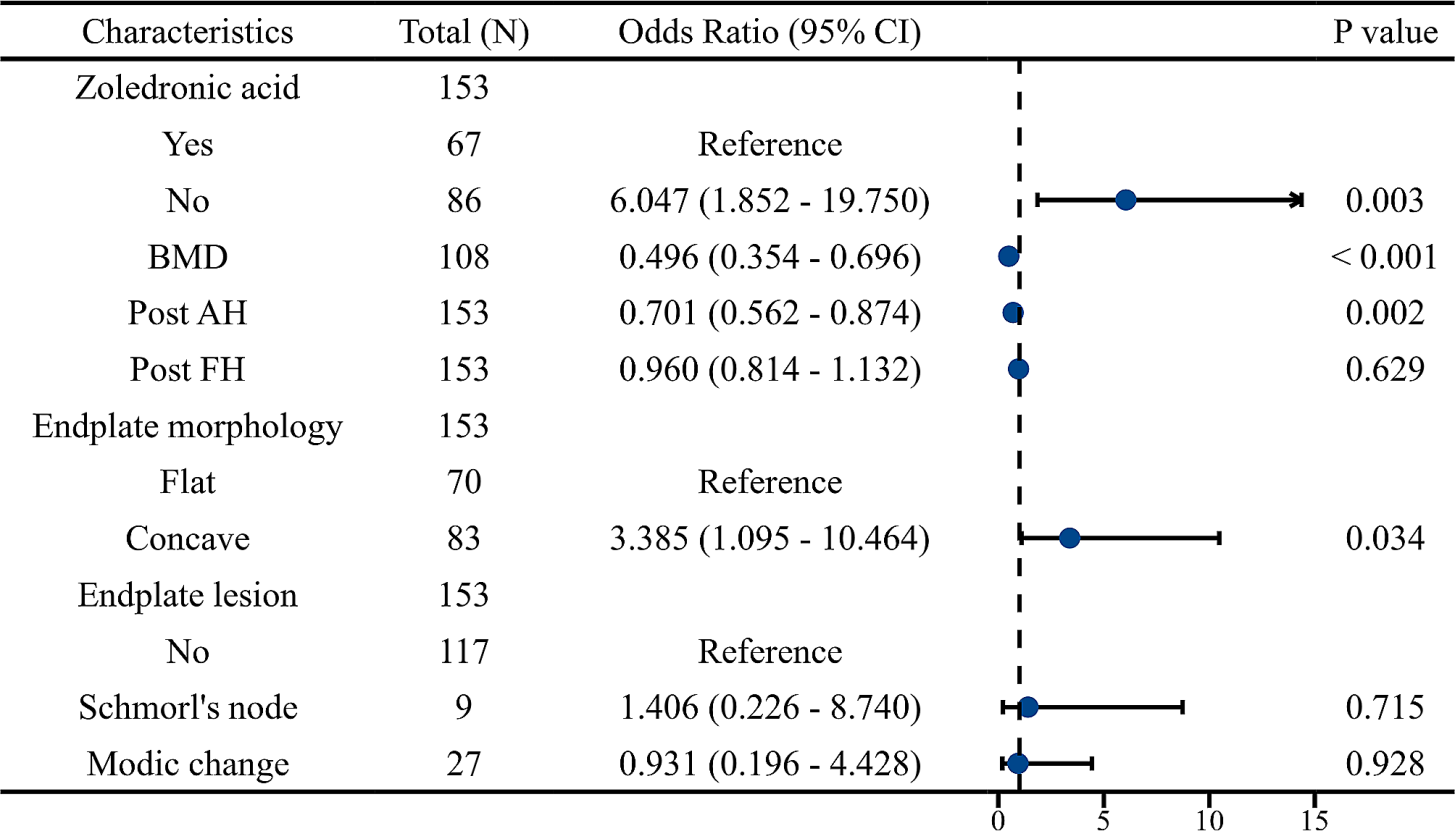
Multivariate analysis of risk factors for cage subsidence after OLIF combined with posterior fixation. BMD: bone mineral density; AH: anterior height; FH: foraminal height

**Fig. 5:**
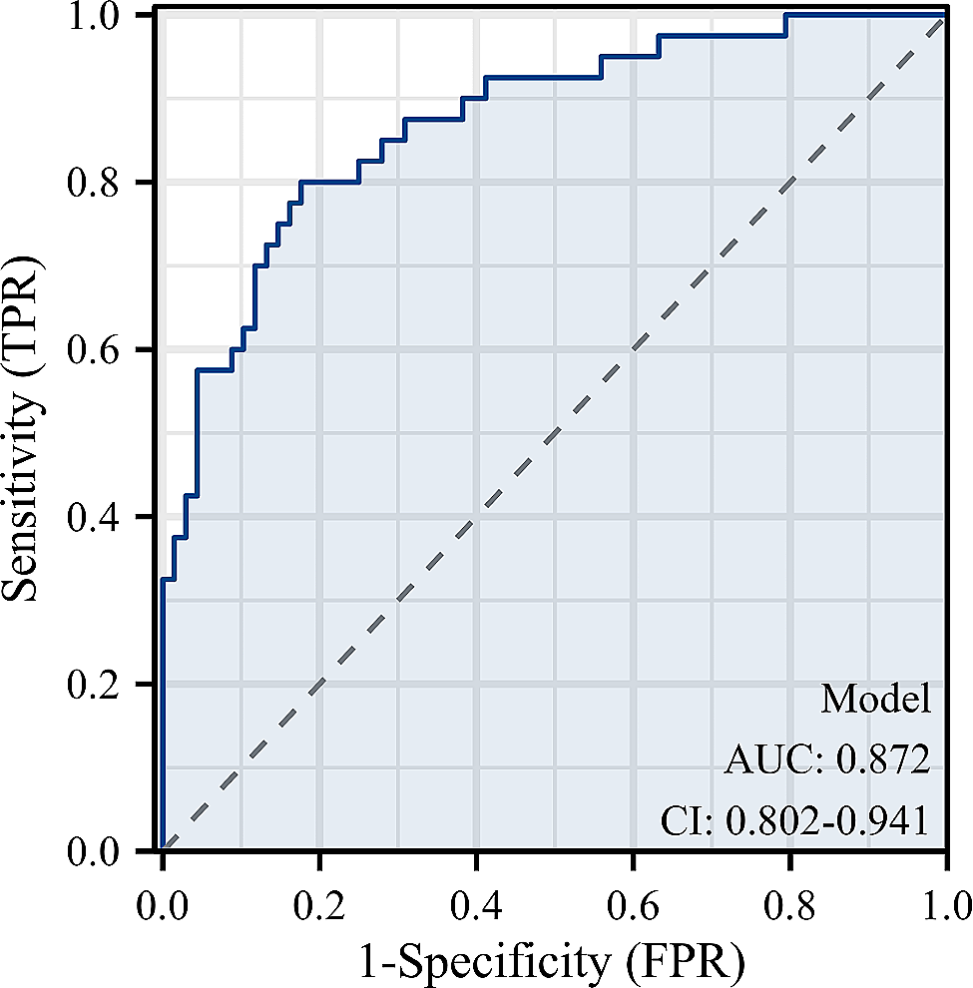
Receiver operating curve. Receiver operating characteristic curve demonstrating the accuracy of the model for predicting cage subsidence after OLIF combined with bilateral pedicle screw fixation. The area under the curve (AUC) was 0.872 (95% CI 0.802–0.941)

According to the multivariate analysis, risk factors independently associated with cage subsidence after OLIF combined with bilateral pedicle screw fixation included no use of zoledronic acid (OR = 6.047, 95% CI 1.852–19.750; *P* = 0.003), lower BMD (OR = 0.496, 95% CI 0.354–0.696; *P* < 0.001), lower postoperative AH (OR = 0.701, 95% CI 0.562–0.874; *P* = 0.002), and concave endplate morphology (OR = 3.385, 95% CI 1.095–10.464; *P* = 0.034) (Fig. 4). The area under the curve (AUC) was 0.872 (95% CI 0.802–0.941) (Fig. 5).

## Discussion

In this study, our most significant finding was that zoledronic acid can reduce the rate of cage subsidence after OLIF combined with bilateral pedicle screw fixation in elderly patients. This is also the first report in the literature on the role of zoledronic acid combined with OLIF. The cage subsidence rate in the Zol group was 20.9%, which was significantly lower than that in the control group (43.0%). Moreover, at 1 year postoperatively, the interbody fusion rates were 93.0% in the Zol group and 90.8% in the control group, with no significant difference. Univariate and multivariate regression analyses revealed that postoperative nonuse of zoledronic acid was a significant risk factor for cage subsidence.

Cage subsidence represents a common complication following OLIF surgery and can adversely affect the efficacy of indirect decompression, potentially leading to the recurrence of neurological symptoms and necessitating reoperation [5]. Hence, it is crucial to identify personalized risk factors for cage subsidence after OLIF surgery and develop corresponding preventive measures. Despite previous studies reporting on risk factors for cage subsidence following OLIF surgery, comprehensive evaluations in the literature are lacking. For instance, in a retrospective analysis of 107 OLIF surgery patients, high-risk factors for cage subsidence were identified, including age over 60 years, BMD less than − 2.5, higher cage height, and severe paravertebral muscle degeneration [15]. Similarly, Hu Z et al. suggested a correlation between vertebral endplate morphology and cage subsidence, with flat endplates and smaller concave angles reducing the likelihood of subsidence [21]. Additionally, Wu H et al. proposed that low Hounsfield unit values are high-risk factors for cage subsidence [22]. Our study comprehensively evaluated and analyzed risk factors for cage subsidence post-OLIF with posterior fixation, identifying lower BMD, lower postoperative anterior disc space height, and concave endplate morphology as high-risk factors.

Compared to other lumbar fusion techniques, OLIF has a relatively greater incidence of cage subsidence postoperatively. Hiyama A reported a 33.9% cage subsidence rate in a retrospective analysis of 59 single-level lateral lumbar interbody fusion cases [23], while Alan N reported only an 8% subsidence rate [24]. Conversely, Kotheeranurak V reported a 46.7% subsidence rate among 107 OLIF surgery patients [15], and Zhao W reported a 62.4% subsidence rate in 85 patients who underwent standalone OLIF surgery [25]. Despite efforts to mitigate the risk of subsidence with OLIF combined with posterior fixation, the incidence of this condition remains high. Parisien A’s systematic review of 245 OLIF patients revealed an average subsidence rate of 17.6%, reaching 36.9% in some cases [26]. Bilateral pedicle screw fixation is considered optimal for preventing subsidence, as supported by biomechanical studies [27]. Hiyama A’s randomized controlled study revealed significantly lower subsidence rates with bilateral fixation [28]. Similarly, Wen J reported 38.9% subsidence in the bilateral group versus 47.3% in the unilateral group [6]. In our study of 108 OLIF patients who underwent bilateral pedicle screw fixation, we observed a 33.3% subsidence rate, consistent with prior research.

Previous reports have shown that zoledronic acid does not increase the interbody fusion rate following posterior lumbar spine fusion surgery. Guppy KH et al. studied 1040 patients, including 632 osteoporotic and 408 osteopenia patients, and found no enhancement in fusion rates with preoperative bisphosphonate use [10]. Li C et al. conducted a randomized controlled trial with 82 patients, yielding similar results [11]. Systematic reviews by Mei J and Fretes N also concluded that postoperative bisphosphonate use did not affect fusion rates [12, 29]. In our study, which focused on elderly patients who underwent OLIF combined with posterior fixation, the postoperative application of zoledronic acid did not increase the interbody fusion rate at 1 year after surgery. The fusion rates were 93.0% in the ZOL group and 90.8% in the control group, with no significant difference. We reviewed the literature on the application of zoledronic acid in transforaminal lumbar interbody fusion (TLIF) and reported that the fusion rates ranged from 70 to 88.5% in the ZOL group and from 56 to 85.5% in the control group [7, 11, 30]. In comparison, the fusion rate in our study was significantly greater than that reported for TLIF surgeries. Additionally, the interbody fusion rates after OLIF surgery at one year postoperatively ranged from 91.2–95.7% [13, 15, 31], which is consistent with the results of our study. Variations in patient characteristics, surgical techniques, and postoperative care protocols may explain differences in fusion rates. Further investigation into these factors is warranted to understand the disparities between our study and previous reports on OLIF.

Our study provides evidence supporting the role of zoledronic acid in reducing the risk of cage subsidence following OLIF surgery, particularly as the first reported evidence of the efficacy of zoledronic acid in treating degenerative lumbar diseases in elderly patients undergoing OLIF. Previous literature also suggests that bisphosphonates may decrease the risk of subsidence after posterior lumbar fusion. For instance, Tu CW et al. reported a 28% subsidence rate at 2 years post-TLIF in the ZOL group compared to 54% in controls [7]. Systematic reviews and meta-analyses by Mei J and Buerba RA et al. also indicated reduced subsidence, vertebral fractures, and pedicle screw loosening with postoperative bisphosphonate use [12, 32]. In our study, the ZOL group had a significantly lower subsidence rate (20.9%) than the control group (43.0%). Multivariate regression analysis revealed no use of zoledronic acid to be an independent risk factor for subsidence (OR = 6.047, *P* = 0.003).

Zoledronic acid reduces cage subsidence risk after OLIF surgery through various mechanisms. As a potent inhibitor of bone resorption, it enhances bone density by inhibiting osteoclast activity [7], providing better cage support and reducing subsidence risk. Additionally, zoledronic acid may improve bone quality by promoting structurally sound bone tissue formation [32] and enhancing intervertebral segment stability. Our study did not show a significant postoperative increase in BMD in the ZOL group, which differs from previous literature reports [30, 33]. Several potential factors may contribute to this difference, including patient population characteristics, comorbidities, sample size, follow-up duration, surgical techniques, and postoperative rehabilitation protocols [30, 33]. The anti-inflammatory properties of zoledronic acid may also contribute to reduced subsidence risk by promoting fusion and integration of the cage with surrounding bone [34]. Furthermore, it helps prevent bone loss around the fusion site, mitigating the risk of subsidence associated with weakened bone structure due to reduced mechanical loading and immobilization. However, further research is needed to fully elucidate the specific mechanisms involved and to optimize its clinical application.

Our study has several limitations. First, the retrospective nature of the study may introduce inherent biases and limitations, including selection bias and information bias. The lack of randomization and blinding could impact the validity of the findings and the interpretability of the results. Additionally, the duration of follow-up in the study may have been relatively short to capture long-term outcomes and complications associated with OLIF combined with posterior fixation and zoledronic acid use. Longer follow-up periods are necessary to assess the durability and sustainability of the observed effects. Finally, this study focused specifically on elderly patients, did not target individuals with osteopenia or osteoporosis. The findings may not be applicable to younger patients, or individuals with specific comorbidities that were not represented in the study population. Although matching based on factors such as age and preoperative BMD was not conducted, baseline characteristics were comparable between the two groups. Future studies with larger sample sizes, longer follow-up periods, randomized controlled designs, and multicenter collaborations may help overcome these limitations and provide more robust evidence regarding the role of zoledronic acid in reducing cage subsidence post-OLIF surgery.

## Conclusion

The administration of zoledronic acid mitigates the risk of cage subsidence following OLIF combined with bilateral pedicle screw fixation in elderly patients; however, it does not improve the interbody fusion rate.

### Electronic supplementary material

Below is the link to the electronic supplementary material.

Supplementary Material 1

Supplementary Material 2

Supplementary Material 3

## Data Availability

The datasets generated during and/or analyzed during the current study are available from the corresponding author upon reasonable request.

## Abbreviations

OLIF: oblique lumbar interbody fusion
ZOL: zoledronic acid
AUC: area under the curve
BMI: body mass index
BMD: bone mineral density
AH: anterior height of the intervertebral space
PH: posterior height of the intervertebral space
DH: intervertebral disc height
CDA: coronal intervertebral disc angle
SDA: sagittal intervertebral disc angle
FH: foraminal height
LL: lumbar lordosis
PACS: Picture Archiving and Communication System
VAS: visual analog scale
ODI: Oswestry Disability Index
SD: standard deviation
IQR: interquartile range
TLIF: transforaminal lumbar interbody fusion
