# Supplementary material for: Can zoledronic acid reduce the risk of cage subsidence after oblique lumbar interbody fusion combined with bilateral pedicle screw fixation in the elderly population? A retrospective study
Source: J Orthop Surg Res. 2024 Jun 8;19:344. doi: 10.1186/s13018-024-04828-3 (PMC11162006; doi:10.1186/s13018-024-04828-3)
Supplement: Supplementary file 1 — Supplementary Material 1 [file 13018_2024_4828_MOESM1_ESM.docx]

Supplementary table 1. Comparison of preoperative BMD between the two groups.

| **Characteristics** | **ZOL group** | **Control group** | **P value** |
| --- | --- | --- | --- |
| N | 43 | 65 |  |
| Preoperative BMD, median (IQR) | -0.7 (-1.9, 1.05) | -1 (-2.6, 1.1) | 0.308 |
| BMD, n (%) |  |  | 0.134 |
| T≧-1.0 | 12 (11.1%) | 14 (13%) |  |
| -2.5≦T﹤-1.0 | 5 (4.6%) | 18 (16.7%) |  |
| T﹤-2.5 | 26 (24.1%) | 33 (30.6%) |  |
