# Supplementary material for: Can zoledronic acid reduce the risk of cage subsidence after oblique lumbar interbody fusion combined with bilateral pedicle screw fixation in the elderly population? A retrospective study
Source: J Orthop Surg Res. 2024 Jun 8;19:344. doi: 10.1186/s13018-024-04828-3 (PMC11162006; doi:10.1186/s13018-024-04828-3)
Supplement: Supplementary file 2 — Supplementary Material 2 [file 13018_2024_4828_MOESM2_ESM.docx]

**Supplementary Table 2. Comparison of the severity of cage subsidence between the two groups.**

|  | ZOL | Control | P value |
| --- | --- | --- | --- |
| Grade, n (%) |  |  | 0.485 |
| 0 | 14 (27.5%) | 33 (64.7%) |  |
| 1 | 0 (0%) | 4 (7.8%) |  |
| 2 | 0 (0%) | 0 (0%) |  |
| 3 | 0 (0%) | 0 (0%) |  |
