# Supplementary material for: Can zoledronic acid reduce the risk of cage subsidence after oblique lumbar interbody fusion combined with bilateral pedicle screw fixation in the elderly population? A retrospective study
Source: J Orthop Surg Res. 2024 Jun 8;19:344. doi: 10.1186/s13018-024-04828-3 (PMC11162006; doi:10.1186/s13018-024-04828-3)
Supplement: Supplementary file 3 — Supplementary Material 3 [file 13018_2024_4828_MOESM3_ESM.pdf]

Dear editor:

We sincerely appreciate the opportunity to improve this manuscript. Firstly, we extend our gratitude for providing us with the chance to enhance our article and for the constructive comments provided by the editor regarding our manuscript titled "Can Zoledronic Acid Reduce the Risk of Cage Subsidence after Oblique Lumbar Interbody Fusion Combined with Bilateral Pedicle Screw Fixation in the Elderly Population? A Retrospective Study." We value and consider all these comments to be valuable and helpful for refining our work.

All authors have thoroughly deliberated on these comments, and in accordance with the reviewers' feedback, we have made every effort to revise our manuscript to align with the requirements of your esteemed journal. In this revised version, all modifications within the document have been highlighted using red text for clarity.

Following this letter, you will find point-by-point responses to the editor’s comments.

Thank you once again for your guidance and support throughout this revision process.

1. Comment: *Is the study design appropriate to answer the research question (including the use of appropriate controls), and are the conclusions supported by the evidence presented?*

Response: I appreciate the editor's question regarding the appropriateness of our study design and the support for our conclusions. In this study, our study population comprised patients aged over 50 with degenerative lumbar diseases treated with OLIF combined with posterior fixation, grouped based on postoperative zoledronic acid use. Although we did not match based on factors such as age and preoperative BMD, baseline characteristics including age, gender, BMD, surgical segments, and number of surgical segments showed no statistically significant differences between the Zol group and the control group, indicating comparability between the two groups. Of course, the editor's suggestions also provide direction for our future research endeavors.

The conclusions drawn from our study are supported by the evidence presented. We found a significant reduction in cage subsidence rates in the zoledronic acid group compared to the control group, highlighting the potential of zoledronic acid in mitigating this complication following OLIF surgery. However, we also acknowledge that the administration of zoledronic acid did not result in a significant increase in interbody fusion rates. In conclusion, while our study provides valuable insights into the role of zoledronic acid in OLIF surgery, we recognize the limitations inherent in our

retrospective design and the need for further research to validate our findings.

Change: (Page 22, Line 418-420) Although matching based on factors such as age and preoperative BMD was not conducted, baseline characteristics were comparable between the two groups.

2. Comment: *The use of the 2mm threshold to mark subsidence is interesting. However other metrics could be employed to further investigate the severity of subsidence.*

Response: I appreciate the editor's observation regarding the threshold used to define subsidence in our study. The choice of a 2mm threshold for defining subsidence is one approach among several possible metrics, as acknowledged. While our study employed this threshold for consistency and comparability with existing literature[1-3], we recognize that alternative metrics could offer additional insights into the severity of subsidence. Notably, previous literature has reported grading the degree of cage subsidence based on the reduction in disc height (DH) postoperatively, with different grades representing varying degrees of disc height loss[4, 5].

Following the editor's suggestion and in accordance with previous literature, an additional analysis comparing the severity of cage subsidence between the two groups was conducted (Supplementary Table 2). The results indicated no statistically significant differences between the groups, and none of the patients experienced severe cage subsidence (graded as Grade 2 and 3). This observation may be attributed to our study population, consisting of patients treated with OLIF combined with bilateral pedicle screw fixation, which may substantially reduce the risk of severe cage subsidence post-OLIF. However, further validation in future studies is warranted.

Supplementary Table 2. Comparison of the severity of cage subsidence between the two groups.

	**ZOL**	**Control**	**P value**
Grade, n (%)			0.485
0	14 (27.5%)	33 (64.7%)	
1	0 (0%)	4 (7.8%)	
2	0 (0%)	0 (0%)	
3	0 (0%)	0 (0%)	

Future research exploring alternative thresholds or complementary measures to evaluate the extent and impact of subsidence would be beneficial in enhancing our understanding of this complication following OLIF surgery. Thank you for highlighting this aspect, and we will consider incorporating alternative metrics in future studies to provide a more comprehensive assessment of subsidence severity.

1. Chung NS, Lee HD, Jeon CH: The Impact of Vertebral End Plate Lesions on the Radiological Outcome in Oblique Lateral Interbody Fusion. *Global Spine J* 2021, 11(8):1176-1182.
2. Park MK, Kim KT, Bang WS, Cho DC, Sung JK, Lee YS, Lee CK, Kim CH, Kwon BK, Lee WK *et al*: Risk factors for cage migration and cage retropulsion following transforaminal lumbar interbody fusion. *Spine J* 2019, 19(3):437-447.
3. Xie T, Pu L, Zhao L, Lu Y, Yang Z, Wang X, Song Y, Zeng J: Influence of coronal-morphology of endplate and intervertebral space to cage subsidence and fusion following oblique lumbar interbody fusion. *BMC Musculoskelet Disord* 2022, 23(1):633.
4. Ma X, Lin L, Wang J, Meng L, Zhang X, Miao J: Oblique lateral interbody fusion combined with unilateral versus bilateral posterior fixation in patients with osteoporosis. *J Orthop Surg Res* 2023, 18(1):776.
5. Marchi L, Abdala N, Oliveira L, Amaral R, Coutinho E, Pimenta L: Radiographic and clinical evaluation of cage subsidence after stand-alone lateral interbody fusion. *J Neurosurg Spine* 2013, 19(1):110-118.

3. Comment: Maybe the inclusion of a survival analysis to assess whether in time each group presented with subsidence would be nice.

Response: I appreciate the editor's suggestion regarding the potential inclusion of a survival analysis to assess the timing of subsidence occurrence in each group. Survival analysis could indeed offer valuable insights into the temporal patterns of subsidence development following OLIF surgery. We acknowledge the merit of this approach in providing a more nuanced understanding of the dynamics of cage subsidence over time. Incorporating survival analysis could enhance our ability to discern differences in the timing and frequency of subsidence events between groups, thereby contributing to a deeper comprehension of this postoperative complication. However, in this retrospective study, we only assessed cage subsidence at 1 year postoperatively, the data on cage subsidence evaluations at 1 month, 3 months, or 6 months postoperatively were not available. We will carefully consider the feasibility and

potential benefits of incorporating survival analysis into our future research endeavors. Thank you for highlighting this suggestion, and we will explore its applicability in further enhancing the robustness of our study findings.

4. Comment: Further investigating the evolution of the subsidence could be interesting in the present study (Ex: patient needed reoperation, or, delta subsidence from each time point to asses if the endplate damage is increasing or decreasing).

Response: I appreciate the editor's insightful suggestion to further investigate the evolution of subsidence in our study. Assessing factors such as the need for reoperation and analyzing the change in subsidence over time, specifically examining delta subsidence at different time points, could indeed provide valuable insights into the progression of endplate damage following OLIF surgery.

These additional analyses could offer a more comprehensive understanding of the long-term implications of cage subsidence and its impact on clinical outcomes. We will carefully consider incorporating these aspects into our future research endeavors to enrich the depth of our findings.

Thank you for highlighting these avenues for further exploration, and we will explore their feasibility in enhancing the comprehensiveness of our study.
